# Changes in basic composition and *in vitro* digestive characteristics of pork induced by frozen storage

**DOI:** 10.3389/fnut.2024.1511698

**Published:** 2025-01-08

**Authors:** Rui Wang, Yongqing Liu, Ying He, Caiping Feng, Xiufang Xia

**Affiliations:** ^1^Department of Biological and Food Engineering, Lyuliang University, Lvliang, Shanxi, China; ^2^College of Food Science, Northeast Agricultural University, Harbin, Heilongjiang, China

**Keywords:** basic composition, digestive characteristics, pork, frozen storage, *in vitro*

## Abstract

**Introduction:**

Frozen pork can reduce the quality of the meat and alter the digestibility and bioavailability of meat proteins in the human body. In this study, we investigated the changes in the basic composition during frozen storage and their effects on the structural properties of digestion products after protein digestion.

**Methods:**

The impacts of frozen storage at different temperatures (−8, −18, −25, and −40°C) and for different times (1, 3, 6, 9, and 12 months) on the basic components and *in vitro* digestive characteristics of pork were evaluated.

**Results:**

The moisture, crude fat, and protein contents decreased with extended storage and increased temperature, whereas muscle juice loss increased (*p* < 0.05). During *in vitro* digestion of samples frozen at −8°C for 12 months, trichloroacetic acid (TCA)-soluble peptides were decreased by 25.46% and 14.37% in the gastric and small intestinal phases, respectively, compared with fresh samples. Confocal laser scanning microscope (CLSM) showed that samples stored at −8°C had the largest particle size after digestion. Disruption of protein structure was confirmed by the decrease in α-helix, β-turn, and fluorescence intensity (all *p* < 0.05) and the increase in β-sheet, random coil, and maximum fluorescence wavelength of the digestion products of samples frozen at −8°C (all *p* < 0.05).

**Discussion:**

Therefore, long-term high-temperature frozen storage brought about a significant decline in basic components of muscle and acceleration of loss of protein structural integrity after digestion.

## 1 Introduction

Pork is a major meat product in the daily diet. It is rich in nutrients, such as high protein and water, polyunsaturated fatty acids, minerals, and easy digestion (1). In order to retain the digestibility and nutritional quality of meat to the maximum extent, freezing is a simple and practical storage method for preserving freshness and extending the shelf life of muscle products (2). Freezing can restrain the growth and propagation of microbial populations and the biological activity of enzymes (3).

Water, protein, and fat are the most basic and important components of pork muscle. Water is the most abundant component of meat products and accounts for approximately 65–80% of the total muscle (4). The relative content of water is related to the color, tenderness, and juiciness of muscle products, which directly affects the processing characteristics and preservation stability of the products (5) and determines the quality and shelf life of the final products (6). Protein is another vital and indispensable element in pork (7). Amino acids serve as the basic building blocks of proteins, and the protein content and amino acid composition are crucial criteria for assessing muscle food quality (8). Fat confers the muscle with good taste, juiciness, and flavor (9). However, during the freezing process and frozen storage, water in muscle tissue freezes into ice crystals. As the freezing time is prolonged, the volume and destruction impact of the ice crystals on the cells increase, exacerbating the loss of water from the cells due to disruption of the structural integrity of muscle protein, which reduces the water binding capacity of proteins (10). The loss of water can give rise to tough, less juicy, and indigestible meat (11). In addition to the structural changes, protein and fat in muscle are susceptible to reactive oxygen species, leading to free radical lipid and protein oxidative reactions (12). The formation of oxidation products can result in protein polymerization and cross-linking, which deteriorates the meat quality and changes the meat protein digestibility and availability in the human body (2).

In recent years, the health effects of meat products have attracted increasing attention from consumer. In addition to the sensory aspects of food, consumer tend to choose food based on nutritional quality. Some studies have shown that frozen meat muscle, such as chicken meat and mirror carp (*Cyprinus carpio L*.), can affect the digestion properties and bio-availability of proteins during transit through the gastrointestinal tract, which has a certain impact on the nutritional value (13, 14). The digestibility and absorption of muscle in the gastrointestinal are key factors that decide the nutritional quality of meat (15). Moisture, crude protein, and crude fat are the basic components of meat muscle. Meat product digestion and absorption by the gastrointestinal tract enable the utilization of this basic component by the human body (16, 17). Thus, their relative content directly or indirectly influences the digestive property of the pork protein (18).

Most research on protein digestion *in vitro* has focused on determining the degree of protein digestibility and the size of the particles after digestion (19–21). There are few studies on the structural characteristics of digestive products after frozen pork protein digestion. Thus, this experiment investigated the changes in the basic composition and amino acids of pork during frozen storage and their effects on the structural properties of digestion products after protein digestion.

## 2 Materials and methods

### 2.1 Materials

The longissimus muscles (lumborum) were obtained from six pig carcasses of approximately 24 weeks of age within 24 h after slaughter from a market and transported to the lab in an ice box with the temperature at around 2–4°C. After removing the excessive fat and connective tissue, fresh pork was cut into 24 pieces, and each sample was weighed (90 ± 5 g). All samples were packed in sealed bags. The total pieces were divided into four batches of six pieces each and stored at four different temperatures (−8, −18, −25, and −40°C) for up to 1 year, with samples withdrawn at 0, 1, 3, 6, 9, and 12 months. The relative humidity of storage environment is about 60–70%. Samples were removed from frozen storage at the indicated times and thawed at 4°C for 12 h until the core temperature reached 4°C. In addition, the muscles (90 ± 5 g) were divided into two chops. Chop A (20 ± 5 g) was used to measure the basic composition and amino acid composition of pork. Chop B (70 ± 5 g) was used for simulated protein digestion *in vitro*. The muscles stored for 0 months were analyzed directly.

### 2.2 Preparation of pork myofibrillar protein

The extraction method of MP by Xia et al. (22) was adopted in this study. The extraction solution was mainly composed of extract and lotion. The extract solution was composed of 10 mM phosphate, 0.1 M NaCl, 2 mM MgCl_2_, and EGTA (pH 7.0). The lotion was 0.1 M NaCl. The extraction process was conducted at 4°C. Meat samples (100 g) were added with four times the volume of extraction solution, homogenized (10,000 rpm, 1 min), and refrigerated under agitation (3,500 rpm, 4°C, 15 min). Then, the supernatant was discarded, and the above operation was repeated, extracted three times, then replaced the lotion for two times of homogenization, and the pH was adjusted to 6.0 with 0.1 M HCl after the third homogenization. The supernatant was removed by centrifugation. The precipitate was MP, and the protein concentration was determined by the biuret method.

### 2.3 Determination of basic composition of pork

#### 2.3.1 Moisture content

The moisture content was measured with reference to the Chinese Standard GB 5009.3–2016. The sample (1–2 g) was wrapped with filter paper and placed in an aluminum box. The aluminum box was placed in an oven at 105°C until the samples reached constant weight. The samples were cooled to room temperature, and the aluminum box was re-weighed. Analyses were done in triplicate. The calculation formula is as follows:


$$X=\frac{m_{1}-m_{2}}{m_{1}-m_{3}}\times 100$$

where *m*_1_ and *m*_2_ are the weights of the total mass of the aluminum box and sample before and after drying, respectively, and *m*_3_ is the mass of the aluminum box.

#### 2.3.2 Crude protein content

The crude protein content was determined based on the Chinese Standard GB/T 5009.5–2016. The samples (0.5–1.0 g) were put in the digestive tube, and the catalyst (6.0 g of potassium sulfate and 0.4 g of copper sulfate) was mixed with 12 mL H_2_SO_4_. The mixture was digested at 360 ~ 410°C for 2 h, then distilled until the digestive liquid was clarified. It was heated for 0.5 h, cooled, and then distilled water was added to 100 mL. Afterward, 40 mL of NaOH solution (40%) and boric acid were added and mixed. The mixture was distilled for 5 min. Finally, the boric acid solution was titrated with standard HCl solution (0.05 mol/L). Analyses were done in triplicate. The calculation formula is as follows:


$$X=\frac{\left(V_{1}-V_{2}\right)\times c\times 0.014}{m\times V_{3}/100}\times F\times 100$$

where *V*_1_ and *V*_2_ are the volumes of HCl standard solution consumed by the sample and reagent blank (mL), respectively, *V*_3_ is the volume of digested liquid (mL), *c* is the concentration of HCl standard solution (mol/L), m is the mass of the sample (g), *F* is the coefficient of nitrogen conversion (6.25), and 0.014 is the milliequivalent weight of nitrogen.

#### 2.3.3 Crude fat content

The crude fat content was measured as described by Chinese Standard GB/T 1215009.6–2016. The accurately weighed meat samples (2–5 g) were placed in a Soxhlet extractor, followed by petroleum ether (boiling range: 30–60°C). The distilling flask was heated in a 45°C water bath and extracted for 8–12 h. Then, the samples were dried at 100–105°C for 0.5 h and weighed. Analyses were done in triplicate. The calculation formula is as follows:


$$X=\frac{m_{4}-m_{5}}{m_{4}}\times 100$$

where *m*_4_ is the mass of the dried sample (g), and *m*_5_ is the mass of the extracted and subsequently dried sample (g).

#### 2.3.4 Juice loss

Juice loss was estimated following the method of Sun et al. (23). The samples were removed from frozen storage and weighed before thawing, then hung with wire and thawed at 4°C. Once the core temperature of the meat was 4°C, the meat surface moisture was absorbed by filter paper and then weighed. Analyses were done in triplicate. The calculation formula is as follows:


$$X=\frac{m_{6}-m_{7}}{m_{6}}\times 100$$

where *m*_6_ is the mass of the sample before thawing (g), and *m*_7_ is the mass of the sample after thawing (g).

### 2.4 Determination of amino acid composition and content

Amino acid compositions of pork were measured according to Jo et al. (24). The meat (1 g) was thoroughly mixed with 15 mL of diluted HCl (6 M) for 24 h and filtered. The filtrate was concentrated on a rotary evaporator at 55°C. The residue was collected and thoroughly mixed with 10 mL of sodium citrate buffer (0.2 M, pH 2.2). The amino acid composition of the filtrate was determined using an LA-8080 automatic amino acid analyzer (Hitachi, Japan). The amino acid analyzer uses standard products for instrument calibration to ensure the accuracy and precision of the instrument. The standard reserve liquid of mixed amino acids and mixed amino acid standard working solution was prepared, the detail experimental steps was as follows: accurately weigh a single amino acid standard (accurate to 0.00001 g) in the same beaker, dissolve it with 8.3 mL hydrochloric acid (6 mol/L) solution, transfer it precisely to a 250 mL volumetric bottle, dilute the volume with water to the scale, and mix well. And then, accurately absorb 1.0 mL of the standard reserve liquid of mixed amino acids into a 10 mL volume bottle, add pH 2.2 sodium citrate buffer solution to the scale, and mix well to form the standard serving liquid. Analyses were done in triplicate.

### 2.5 *In vitro* digestion model of pork MP

*In vitro* digestion of MP was assayed by referring to the procedure of Pan et al. (1). The digestion process proceeded as two-stage hydrolysis by pepsin and trypsin. Samples were collected after pepsin and trypsin digestion.

Simulated gastric digestion. The extracted MPs were diluted to 30 mg/mL with a phosphate buffered (PBS: 10 mM Na_2_HPO_4_-NaH_2_PO_4_, pH 7.0). MP solution (10 mL) was mixed with 10 mL of SGF. Pepsin (4,800 U/mL) was dissolved in SGF to catalyze the digestion reaction. The mixture was put in a constant shaking water bath to react for 2 h at 37°C and 200 rpm. After gastric digestion was finished, 1 M of NaOH was used to adjust the pH level of the digested samples 7 to terminate the reaction.

Simulated intestinal digestion. After gastric digestion, 20 mL of pepsin digestive products were added to the 20 mL of SIF. Trypsin (17,940 U/mL) was dissolved in SIF to induce a small intestine digestion phase. The mixture was subjected to shaking under the above mentioned conditions. After 2 h of trypsin digestion, the final reaction was immediately stopped by placing the samples in a boiling water bath for 5 min. Additionally, blank groups (MP solution replaced with distilled water) were used for the whole gastrointestinal digestion procedure.

To inactivate the enzyme from the digested samples, precipitation was carried out using an equal volume of TCA (15%, v/v) at 4°C for 12 h. After that, the supernatant was collected and analyzed via centrifugation (Beckman, United States) with a 10,000 g for 20 min at 4°C.

#### 2.5.1 Determination of TCA-soluble peptides

The TCA-soluble peptides of the digested samples were determined with reference to the method of Buamard and Benjakul (25). Three milliliters of digested sample solution and 27 mL of 5% TCA solution were fully blended. The mixture was homogenized and centrifuged (10,000 g, 4°C, 10 min). The TCA-soluble peptide content was calculated by the folinol method.

#### 2.5.2 Determination of the microstructure of digestive samples

The microstructures of digested products were observed using a previous method by confocal laser scanning microscope (Leica TCS SP5, Heidelberg, Germany; CLSM) (14). Nile blue was used as the fluorescent stain and dissolved in ethanol. After combining 1.0 mL of digested sample solution with 20 μL of fluorescent stain (0.1%, w/v) for 0.5 h, 5 μL was removed and used to capture the morphology of the digested samples (40× objective and 633 nm helium–neon laser).

#### 2.5.3 Determination of the protein secondary structure

The protein secondary structure of the digested products was assayed by Fourier transform infrared (FTIR) spectroscopy. The digested samples were lyophilized. Potassium bromide and freeze-dried digested sample were mixed at a ratio of 10:1, pressed into transparent sheets under a certain pressure, and then placed in the FTIR spectrometer (Perkin Elmer, United States). Spectra were recorded at room temperature in the spectrum range of 400–4,000 cm^−1^, with a resolution of 4 cm^−1^. Each spectrum was an accumulation of 64 scans. OMNIC software was used to process and fit the infrared spectrum of the sample. The percentage of four secondary structures (α-helix, β-sheet, β-turn, and random coil) was estimated by Gaussian fitting of the extracted amide-I spectra (1600–1700 cm^−1^) by using Peak Fit v4.04 software (AISN Software, Inc.).

#### 2.5.4 Determination of the protein tertiary structure

The digested sample solution was adjusted to 1 mg/mL and used to determine the tertiary structure by the method according to Pan et al. (12). An F-7000 Hitachi fluorescence spectrometer (Hi-Tech, Japan) was used, and the scanning parameters were set as follows: emission spectrum range: 300–400 nm, scanning speed: 240 nm/min, excitation wavelength: 280 nm, excitation and emission slit width: 10 nm. The maximum fluorescence intensity (FI) and emission spectra were recorded.

### 2.6 Statistical analysis

All graphs were obtained by the software SigmaPlot 12.5. The General Linear Model (GLM) procedure of the Statistix 8.1 software package (Analytical Software, St Paul, MN, United States) was used to analyze all the data, which were presented as mean ± standard deviation. Each sample was made three times in parallel. Significance was calculated by one-way analysis of variance (ANOVA) and Tukey’s multiple comparisons with a 95% confidence interval (*p* < 0.05).

## 3 Results and discussion

### 3.1 Basic composition

Moisture, crude protein, and crude fat are the most important basic elements of muscle (2). As shown in Table 1, the moisture content showed a significant decline with prolonged freezing storage and increased freezing temperature (*p* < 0.05). After 12 months of frozen storage, the moisture content of samples had decreased to 70.14% (−8°C), 71.15% (−18°C), 72.25% (−25°C), and 72.64% (−40°C). At the same time, the juice loss of samples increased to 6.99% (−8°C), 4.89% (−18°C), 3.49% (−25°C), and 2.33% (−40°C) compared to the unfrozen samples. With prolonged frozen storage, the water in the muscle gradually formed ice crystals that slowly expanded in volume. The higher the freezing temperature, the more irregular shape of ice crystals. The formed irregular ice crystals punctured the muscle cells and damaged the cell structure, resulting in the loss of water and increased juice losses (26). Meanwhile, during the freezing process, the denaturation of protein structure causes the muscle protein to reduce the binding ability of water, so that the water of the muscle cannot be reabsorbed by the cells after thawing, resulting in the loss of water (27). Zhang et al. (28) observed that the freezing process significantly increased the water loss of muscles, ultimately leading to the deterioration of meat quality.

**Table 1:** Influence of frozen temperature (°C) and time (mon) on pork routine nutrients (as 100% meat weight).

Frozen temperature (°C)	Frozen time (mon)	Moisture (%)	Juice loss (%)	Crude protein(%)	Crude fat (%)
−8	0	74.39 ± 0.01^Aa^	–	18.63 ± 0.03^Aa^	4.75 ± 0.02^Aa^
	1	74.21 ± 0.03^Ab^	1.00 ± 0.01^Ea^	18.57 ± 0.03^Aa^	4.70 ± 0.01^Aa^
	3	74.01 ± 0.04^Bc^	1.30 ± 0.02^Da^	18.01 ± 0.02^Bc^	4.64 ± 0.02^Bb^
	6	73.59 ± 0.02^Cc^	2.25 ± 0.03^Ca^	17.55 ± 0.04^BCc^	4.50 ± 0.03^Cb^
	9	71.57 ± 0.02^Dd^	5.24 ± 0.04^Ba^	17.07 ± 0.03^CDc^	4.40 ± 0.06^Dc^
	12	70.14 ± 0.04^Ed^	6.99 ± 0.03^Aa^	16.55 ± 0.04^Dc^	3.98 ± 0.05^Ed^
−18	0	74.38 ± 0.05^Aa^	–	18.64 ± 0.03^Aa^	4.73 ± 0.03^Aa^
	1	74.35 ± 0.02^Aa^	0.79 ± 0.03^Eb^	18.60 ± 0.03^Aa^	4.71 ± 0.02^Aa^
	3	74.15 ± 0.02^Bb^	1.1 ± 0.03^Db^	18.51 ± 0.02^Bbc^	4.69 ± 0.01^Ab^
	6	74.05 ± 0.03^Cb^	1.78 ± 0.02^Cb^	18.15 ± 0.04^Cb^	4.52 ± 0.02^Bab^
	9	72.15 ± 0.01^Dc^	3.81 ± 0.03^Bb^	18.04 ± 0.03^Dc^	4.49 ± 0.02^BCb^
	12	71.15 ± 0.02^Ec^	4.89 ± 0.02^Ab^	18.01 ± 0.04^Db^	4.34 ± 0.04^Cc^
−25	0	74.38 ± 0.02^Aa^	–	18.63 ± 0.02^Aa^	4.74 ± 0.01^Aa^
	1	74.36 ± 0.03^Aa^	0.80 ± 0.02^Eb^	18.61 ± 0.02^Aa^	4.73 ± 0.02^Aa^
	3	74.24 ± 0.03^Ba^	1.01 ± 0.01^Dc^	18.54 ± 0.04^Aab^	4.64 ± 0.02^Bab^
	6	74.14 ± 0.04^Ca^	1.12 ± 0.03^Cc^	18.24 ± 0.03^Bab^	4.58 ± 0.03^BCa^
	9	73.52 ± 0.02^Db^	2.21 ± 0.01^Bc^	18.11 ± 0.04^Cb^	4.51 ± 0.03^CDab^
	12	72.25 ± 0.07^Eb^	3.49 ± 0.01^Ac^	18.05 ± 0.05^Ca^	4.44 ± 0.05^Db^
−40	0	74.39 ± 0.01^Aa^	–	18.63 ± 0.03^Aa^	4.75 ± 0.02^Aa^
	1	74.37 ± 0.01^ABa^	0.55 ± 0.03^Ec^	18.62 ± 0.03^Aa^	4.74 ± 0.02^Aa^
	3	74.31 ± 0.05^Ba^	0.67 ± 0.03^Dd^	18.51 ± 0.03^Ba^	4.71 ± 0.01^ABa^
	6	74.20 ± 0.04^Ca^	0.84 ± 0.03^Cd^	18.41 ± 0.01^Ca^	4.62 ± 0.02^BCa^
	9	73.61 ± 0.05^Da^	1.28 ± 0.02^Bd^	18.21 ± 0.02^Da^	4.54 ± 0.04^CDa^
	12	72.64 ± 0.04^Ea^	2.33 ± 0.03^Ad^	18.11 ± 0.04^Da^	4.49 ± 0.04^Da^

The means at the same frozen temperature with different uppercase letters (A–E) differ significantly (*p* < 0.05); the means at the same frozen time with different lowercase letters (a–d) differ significantly (*p* < 0.05).

After 12 months of frozen storage, the crude protein content of muscle had significantly decreased from 18.63% before storage to 16.55% (−8°C), 18.01% (−18°C), 18.05% (−25°C) and 18.11% (−40°C; *p* < 0.05). This decrease in crude protein content was due to the difference in freezing rate and ice crystal size because the samples had been stored for the same duration. The lower the freezing temperature, the faster the freezing rate, the more uniform the ice crystals formed, the less damage to the cell tissue, and the lower the damage degree of protein (28). Therefore, the protein structure was relatively intact, and the more the crude protein content of the muscle was retained. When the samples were stored at the same temperature, protein oxidation and hydrolysis during frozen storage might lead to a decrease in the crude protein content of muscle (29). Wu et al. (30) showed that during the frozen storage process, small molecules of aldehydes, alcohols, acids and other compounds oxidized by fat will undergo aldehyde-ammonia condensation reaction with the free amino group of proteins, resulting in changes in the original structure of proteins and a corresponding reduction in crude protein content.

After 12 months of frozen storage, the crude fat content of muscle samples was decreased to 3.98% (−8°C), 4.34% (−18°C), 4.44% (−25°C), and 4.49% (−40°C). This decrease in crude fat content might be because of cell muscle tissue puncture by ice crystals, which led to the release of some pro-oxidation factors (oxidizing lipids, free radicals, and heme pigments) and the acceleration of lipid oxidation (29). In addition, intracellular lipase, protease, and nuclease would be released from cells and participate in oxidation reactions, thus accelerating the lipid oxidation reaction and ultimately leading to the decline in crude fat content (31).

### 3.2 Amino acid composition and content

**Figure 1:**
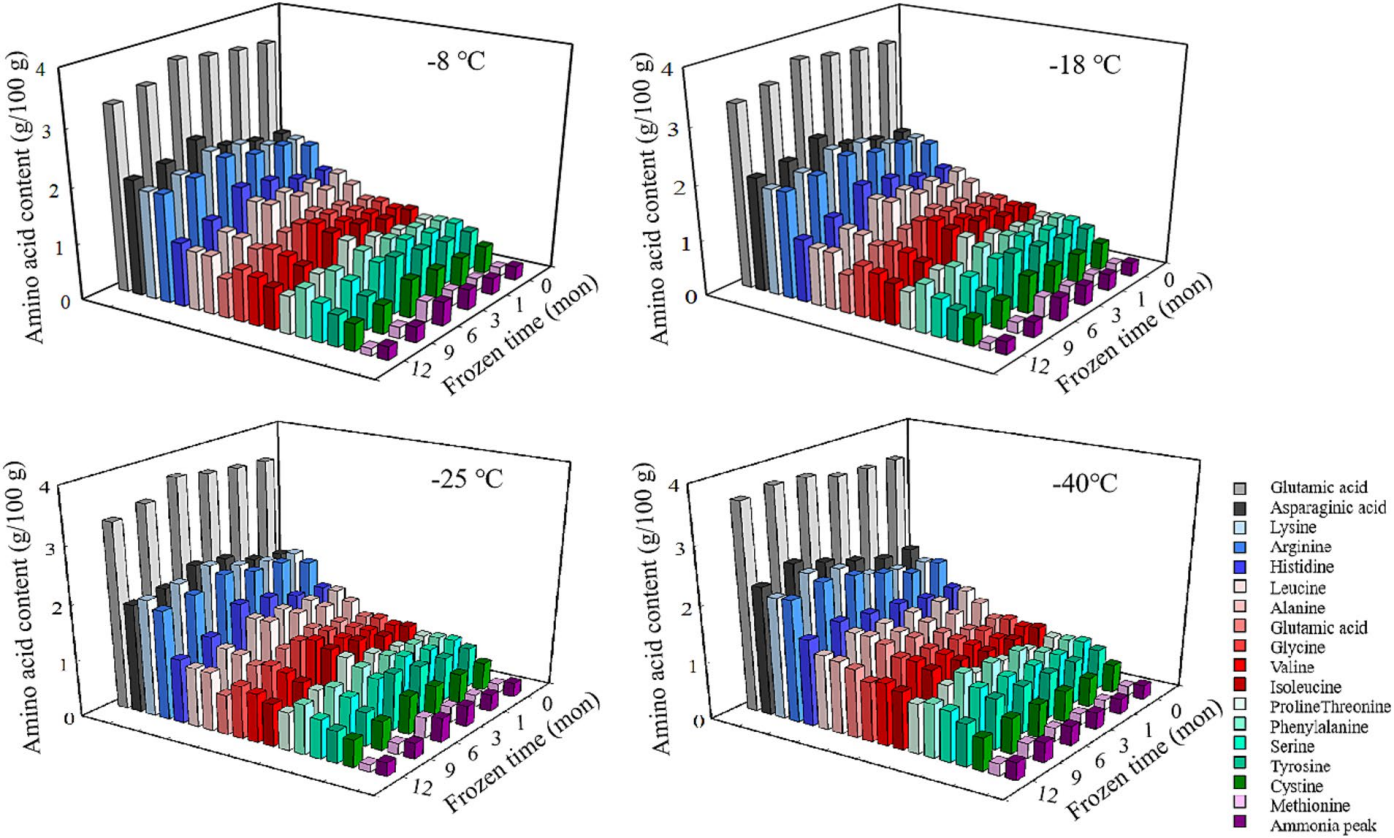
Influence of frozen storage temperature (°C) and time (mon) on the amino acid composition and content of pork.

Amino acids are the basic building blocks of proteins, and the variation of their composition type and relative content determines the diversity of protein structure (8). As shown in Figure 1 and Table 2, there existed 17 types of free amino acids in frozen pork muscle, including 7 essential (Leu, Val, Iso, Thr, Phe, and Met) and 10 non-essential amino acids (Glu, Asp., Arg, His, Ala, Gly, Pro, Tyr, Ser, and Cys). During freezing storage, amino acid types did not change. With the extension of freezing storage time, amino acid contents such as Glu, Asp and Lys showed a trend of first increasing and then decreasing. The higher the freezing storage temperature, the more significant the change of amino acid content was. Among them, Glu was the highest during the frozen storage, followed by Asp., Lys, Arg, and His. Amino acids such as Gly, His, Arg, Ala, Met, Leu, and Phe showed an increasing trend in the first 6 months and then decreased in the following months. For example, the Glu content increased by 6.01% after frozen storage for 6 months and then decreased by 8.02% at 12 months of frozen storage. In addition, the higher the frozen temperature, the higher the free amino acid contents. The increase in amino acid content during the same frozen temperature was due to the partial unfolding of spatial structure induced by mild oxidation of proteins at the beginning of frozen storage, which resulted in the exposure of some amino acids to the protein surface (12). With extended frozen storage, the amino acids gradually underwent oxidation, which resulted in a decline in amino acid content. Bai et al. (3) reported that the increased content of all amino acids during the first four freezethaw cycles could be due to the transition of one amino acid to another amino acid through oxidation and deamination reactions, and with more freezethaw cycles, the decrease in the content of some amino acids might be due to their degradation. When the samples were stored for the same time, the lower the temperature, the lower the degree of protein degradation and the slower the rate of biochemical reactions, thus the higher the content of amino acids (1). Furthermore, the development and expansion of ice crystals throughout the freezing process can pierce the cellular structure of lysozyme, resulting in the discharge of proteolytic enzymes and aminopeptidases, thereby increasing the recognition of amino acids and enzymes of peptide chain segments at specific locations, and ultimately causing changes in amino acid content in muscle (32). Chen et al. (33) determined that the rise in amino acid levels during frozen storage is attributed to an increase in endogenous proteases secreted by microorganisms within the meat, which further catalyzes protein breakdown. The oxidation products are susceptible to the maillard reaction, forming advanced glycation end products (AGEs) and heterocyclic aromatic amines (HAAs) during subsequent processing and storage. These compounds pose risks of chronic diseases such as diabetes and Alzheimer’s disease (34). Additionally, oxidative modifications or aggregation of proteins may mask the binding sites for proteolytic enzymes, thereby affecting the digestibility of proteins (35, 36).

**Table 2:** Influence of frozen temperature (°C) and time (mon) on pork amino acids content (mg/100 g).

Temperature (°C)	Time (mon)	Glu	Asp	Lys	Arg	His	Leu	Ala	Gly	Val	Iso	Pro	Thr	Phe	Ser	Tyr	Cys	Met
−8	0	3.48 ± 0.01^Ca^	1.86 ± 0.01^Ea^	1.71 ± 0.01^Ea^	1.73 ± 0.01^Da^	1.31 ± 0.01^Ca^	1.30 ± 0.01^Ca^	1.14 ± 0.02^Da^	0.93 ± 0.01^Da^	0.93 ± 0.02^Da^	0.87 ± 0.01^Da^	0.90 ± 0.01^Da^	0.78 ± 0.02^Da^	0.80 ± 0.01^Ea^	0.81 ± 0.01^Da^	0.72 ± 0.02^Ca^	0.50 ± 0.00^Ca^	0.16 ± 0.01^Da^
	1	3.52 ± 0.01^Ca^	1.93 ± 0.01^Da^	1.90 ± 0.01^Da^	1.94 ± 0.01^Ca^	1.37 ± 0.01^Ba^	1.34 ± 0.01^Cb^	1.27 ± 0.01^Ca^	1.05 ± 0.02^Ca^	1.01 ± 0.00^Ca^	1.01 ± 0.01^Ca^	0.96 ± 0.01^Ca^	0.83 ± 0.01^Ca^	0.91 ± 0.02^Ca^	0.88 ± 0.01^Ca^	0.75 ± 0.01^Ca^	0.51 ± 0.01^Ca^	0.22 ± 0.01^Ca^
	3	3.61 ± 0.01^Ba^	2.29 ± 0.02^Ba^	2.15 ± 0.02^Ba^	2.00 ± 0.01^Ba^	1.56 ± 0.01^Aa^	1.44 ± 0.01^Ba^	1.38 ± 0.01^Ba^	1.17 ± 0.01^Ba^	1.17 ± 0.01^Ba^	1.11 ± 0.02^Ba^	1.12 ± 0.01^Ba^	0.94 ± 0.02^Ba^	1.08 ± 0.01^Ba^	1.01 ± 0.01^Ba^	0.89 ± 0.01^Ba^	0.59 ± 0.01^Ba^	0.28 ± 0.01^Ba^
	6	3.71 ± 0.01^Ab^	2.39 ± 0.02^Aa^	2.29 ± 0.02^Aa^	2.27 ± 0.01^Aa^	1.60 ± 0.01^Aa^	1.53 ± 0.00^Aa^	1.46 ± 0.02^Aa^	1.35 ± 0.01^Aa^	1.23 ± 0.01^Aa^	1.32 ± 0.01^Aa^	1.23 ± 0.02^Aa^	1.13 ± 0.01^Aa^	1.10 ± 0.01^Aa^	1.04 ± 0.01^Aa^	1.02 ± 0.01^Aa^	0.68 ± 0.01^Aa^	0.36 ± 0.01^Aa^
	9	3.43 ± 0.02^Db^	2.14 ± 0.03^Cc^	2.01 ± 0.00^Cc^	2.00 ± 0.01^Ba^	1.26 ± 0.01^Cc^	1.15 ± 0.01^Dc^	1.10 ± 0.01^Dd^	0.98 ± 0.01^CDd^	1.03 ± 0.01^Cb^	0.98 ± 0.02^Cb^	0.87 ± 0.01^Dc^	0.80 ± 0.01^Cb^	0.91 ± 0.01^Ca^	0.79 ± 0.02^Dc^	0.60 ± 0.01^Dc^	0.51 ± 0.01^Cc^	0.19 ± 0.01^Cb^
	12	3.21 ± 0.02^Ec^	1.95 ± 0.01^Dc^	1.93 ± 0.01^Dc^	1.94 ± 0.00^Ca^	1.13 ± 0.02^Dc^	1.04 ± 0.02^Ec^	1.04 ± 0.01^Ec^	0.71 ± 0.01^Eb^	0.93 ± 0.01^Db^	0.86 ± 0.03^Db^	0.76 ± 0.01^Ec^	0.67 ± 0.02^Eb^	0.88 ± 0.01^Db^	0.69 ± 0.01^Eb^	0.56 ± 0.01^Db^	0.48 ± 0.01^Cb^	0.12 ± 0.01^Dc^
−18	0	3.48 ± 0.01^Ca^	1.88 ± 0.01^Da^	1.71 ± 0.01^Da^	1.73 ± 0.01^Fa^	1.30 ± 0.01^Ca^	1.31 ± 0.01^Ca^	1.13 ± 0.01^Da^	0.91 ± 0.01^Da^	0.93 ± 0.01^Da^	0.86 ± 0.01^Da^	0.90 ± 0.01^Da^	0.77 ± 0.01^Da^	0.79 ± 0.01^Ea^	0.81 ± 0.00^Ca^	0.72 ± 0.02^Ba^	0.49 ± 0.01^Ca^	0.16 ± 0.01^Ea^
	1	3.52 ± 0.01^Ba^	1.91 ± 0.01^Da^	1.77 ± 0.01^Cb^	1.85 ± 0.01^Eb^	1.33 ± 0.02^Cb^	1.40 ± 0.01^Ba^	1.23 ± 0.01^Cb^	0.93 ± 0.01^Db^	0.98 ± 0.01^CDa^	1.01 ± 0.02^Ca^	0.93 ± 0.01^CDa^	0.81 ± 0.01^Ca^	0.93 ± 0.01^Da^	0.84 ± 0.01^Cb^	0.74 ± 0.01^Ba^	0.50 ± 0.01^Ca^	0.21 ± 0.00^CDa^
	3	3.54 ± 0.01^Bbc^	2.17 ± 0.01^Bb^	2.12 ± 0.01^Bb^	1.99 ± 0.01^Ca^	1.56 ± 0.01^Ba^	1.41 ± 0.01^Ba^	1.36 ± 0.01^Ba^	1.15 ± 0.01^Ba^	1.11 ± 0.01^Bb^	1.07 ± 0.01^Ba^	1.04 ± 0.01^Bb^	0.90 ± 0.01^Bb^	0.97 ± 0.01^Cb^	0.94 ± 0.01^Bb^	0.86 ± 0.01^Ab^	0.57 ± 0.01^Ba^	0.26 ± 0.01^Ba^
	6	3.81 ± 0.01^Aa^	2.36 ± 0.03^Aa^	2.26 ± 0.01^Ab^	2.23 ± 0.02^Aa^	1.59 ± 0.01^Aa^	1.45 ± 0.02^Ab^	1.45 ± 0.02^Aa^	1.33 ± 0.02^Aa^	1.22 ± 0.01^Aa^	1.19 ± 0.01^Ab^	1.20 ± 0.01^Aa^	1.11 ± 0.01^Aab^	1.01 ± 0.01^Bb^	1.03 ± 0.01^Aab^	0.89 ± 0.01^Ab^	0.63 ± 0.02^Ab^	0.31 ± 0.01^Ab^
	9	3.41 ± 0.01^Db^	2.32 ± 0.01^Ab^	2.14 ± 0.01^Bb^	2.03 ± 0.01^Ba^	1.31 ± 0.00^Cc^	1.34 ± 0.01^Cb^	1.20 ± 0.01^Cb^	1.04 ± 0.01^Cc^	1.03 ± 0.02^Cb^	1.01 ± 0.02^Cb^	0.93 ± 0.01^Cb^	0.84 ± 0.01^Cb^	1.08 ± 0.01^Ab^	0.84 ± 0.01^Cb^	0.61 ± 0.01^Cc^	0.51 ± 0.01^Cc^	0.23 ± 0.01^BCa^
	12	3.31 ± 0.01^Eb^	2.08 ± 0.02^Cb^	1.94 ± 0.01^Cc^	1.94 ± 0.01^Da^	1.15 ± 0.01^Dc^	1.10 ± 0.01^Db^	1.11 ± 0.03^Eb^	0.71 ± 0.02^Eb^	0.95 ± 0.01^Db^	0.87 ± 0.01^Db^	0.81 ± 0.01^Eb^	0.69 ± 0.01^Eb^	0.89 ± 0.01^Db^	0.72 ± 0.01^Db^	0.58 ± 0.01^Db^	0.51 ± 0.00^Cb^	0.16 ± 0.01^Eb^
−25	0	3.48 ± 0.01^Ca^	1.82 ± 0.01^Fa^	1.71 ± 0.01^Ea^	1.73 ± 0.01^Ea^	1.31 ± 0.02^Ca^	1.30 ± 0.01^Da^	1.14 ± 0.01^Ea^	0.91 ± 0.01^Da^	0.94 ± 0.01^Da^	0.86 ± 0.01^Da^	0.90 ± 0.01^CDa^	0.78 ± 0.01^Da^	0.80 ± 0.01^Ea^	0.80 ± 0.02^Ca^	0.72 ± 0.01^Ca^	0.49 ± 0.01^Ca^	0.16 ± 0.01^Da^
	1	3.5 ± 0.02^Ca^	1.92 ± 0.01^Eb^	1.74 ± 0.02^Dbc^	1.84 ± 0.02^Db^	1.34 ± 0.01^Cb^	1.34 ± 0.01^Cb^	1.21 ± 0.01^Db^	0.92 ± 0.01^Db^	0.96 ± 0.02^Da^	1.00 ± 0.01^Ca^	0.93 ± 0.01^Cab^	0.83 ± 0.01^Ca^	0.92 ± 0.01^Da^	0.83 ± 0.01^Cb^	0.72 ± 0.02^Ca^	0.50 ± 0.01^Ca^	0.21 ± 0.20^Ca^
	3	3.56 ± 0.01^Bb^	2.14 ± 0.01^Bb^	2.05 ± 0.01^Cc^	1.89 ± 0.01^DCb^	1.53 ± 0.01^Ba^	1.40 ± 0.01^Bb^	1.36 ± 0.01^Ba^	0.13 ± 0.02^Ba^	1.17 ± 0.01^Ba^	1.07 ± 0.01^Ba^	1.04 ± 0.01^Bb^	0.90 ± 0.01^Bb^	0.96 ± 0.01^Cb^	0.93 ± 0.01^Bb^	0.84 ± 0.01^Bb^	0.56 ± 0.01^Ba^	0.27 ± 0.01^Ba^
	6	3.8 ± 0.01^Aa^	2.22 ± 0.01^C^	2.25 ± 0.01^Db^	2.14 ± 0.01^Ab^	1.59 ± 0.01^Aa^	1.46 ± 0.01^Ab^	1.40 ± 0.01^Ab^	1.22 ± 0.01^Ab^	1.21 ± 0.02^Aa^	1.20 ± 0.01^Ab^	1.14 ± 0.01^Ab^	1.09 ± 0.01^Abc^	1.00 ± 0.01^Bb^	1.01 ± 0.01^Aa^	0.88 ± 0.01^Ab^	0.63 ± 0.01^Ab^	0.31 ± 0.01^Ab^
	9	3.42 ± 0.01^Db^	2.02 ± 0.01^Dd^	2.14 ± 0.01^Bb^	2.00 ± 0.02^Ba^	1.37 ± 0.03^Cb^	1.35 ± 0.02^Cb^	1.30 ± 0.02^Cb^	1.11 ± 0.02^BCb^	1.08 ± 0.01^Cb^	1.02 ± 0.01^Cb^	0.94 ± 0.02^Cb^	0.93 ± 0.01^Ba^	1.09 ± 0.01^Ab^	0.84 ± 0.01^Cb^	0.66 ± 0.01^Db^	0.57 ± 0.01^Bb^	0.24 ± 0.03^Ba^
	12	3.32 ± 0.01^Eb^	2.09 ± 0.01^Cb^	2.07 ± 0.01^Cb^	1.93 ± 0.02^Ca^	1.20 ± 0.01^Db^	1.12 ± 0.01^Eb^	1.10 ± 0.01^Eb^	0.75 ± 0.01^Eab^	0.96 ± 0.01^Db^	0.88 ± 0.01^Cb^	0.82 ± 0.01^Eb^	0.68 ± 0.02^Eb^	0.90 ± 0.01^Db^	0.70 ± 0.02^Db^	0.61 ± 0.01^Eb^	0.51 ± 0.01^Cb^	0.17 ± 0.01^Db^
−40	0	3.48 ± 0.01^Ca^	1.88 ± 0.01 ^E^	1.70 ± 0.01^Ea^	1.73 ± 0.01^Da^	1.31 ± 0.01^Da^	1.30 ± 0.01^CDa^	1.12 ± 0.02^Da^	0.92 ± 0.00^DEa^	0.94 ± 0.01^Da^	0.87 ± 0.01^Da^	0.90 ± 0.01^Da^	0.77 ± 0.01^Da^	0.79 ± 0.00 ^Da^	0.81 ± 0.01^Da^	0.71 ± 0.01^Da^	0.50 ± 0.00^Da^	0.16 ± 0.01^Ca^
	1	3.49 ± 0.02^Ca^	1.92 ± 0.01^Ea^	1.72 ± 0.02^Ec^	1.75 ± 0.02^Dc^	1.34 ± 0.01^Db^	1.33 ± 0.01^Cb^	1.15 ± 0.01^Dc^	0.93 ± 0.01^DEb^	0.96 ± 0.01^Da^	0.89 ± 0.01^Db^	0.92 ± 0.02^CDb^	0.79 ± 0.02^Da^	0.80 ± 0.01^Db^	0.82 ± 0.01^Db^	0.73 ± 0.01^Da^	0.50 ± 0.01^Da^	0.17 ± 0.01^Cb^
	3	3.51 ± 0.01^Cc^	2.08 ± 0.01^Dc^	1.89 ± 0.01^Dd^	1.91 ± 0.01^Cb^	1.46 ± 0.01^Cb^	1.36 ± 0.01^BCc^	1.24 ± 0.01^BCb^	1.10 ± 0.01^c^	1.05 ± 0.01^Cc^	1.01 ± 0.01^Cb^	0.95 ± 0.01^Cc^	0.84 ± 0.01^Cc^	0.94 ± 0.02^Cb^	0.88 ± 0.01^Cc^	0.80 ± 0.01^Cc^	0.54 ± 0.01^BCa^	0.19 ± 0.01^Bb^
	6	3.67 ± 0.03^ABb^	2.2 ± 0.02^Cb^	2.20 ± 0.01^BCa^	2.13 ± 0.01^Bb^	1.51 ± 0.01^Bb^	1.40 ± 0.00^Bc^	1.29 ± 0.02^Bc^	1.26 ± 0.01^Bb^	1.13 ± 0.02^Bb^	1.08 ± 0.01^Bc^	1.04 ± 0.01^Bc^	0.89 ± 0.01^Bd^	0.99 ± 0.01^Bb^	0.94 ± 0.01^Bb^	0.84 ± 0.01^Bc^	0.58 ± 0.01^Bc^	0.23 ± 0.01^Ac^
	9	3.71 ± 0.01^Ab^	2.43 ± 0.01^Aa^	2.33 ± 0.01^Aa^	2.23 ± 0.01^Ab^	1.60 ± 0.01^Aa^	1.46 ± 0.01^Aa^	1.46 ± 0.01^Aa^	1.38 ± 0.01^Aa^	1.22 ± 0.01^Aa^	1.22 ± 0.01^Aa^	1.12 ± 0.02^Aa^	0.92 ± 0.01^Aa^	1.20 ± 0.01^Aa^	1.01 ± 0.01^Aa^	0.89 ± 0.01^Aa^	0.63 ± 0.01^Aa^	0.25 ± 0.01^Aa^
	12	3.64 ± 0.02^Ba^	2.28 ± 0.02^Ba^	2.12 ± 0.01^Ca^	2.14 ± 0.01^Ba^	1.52 ± 0.01^Ba^	1.31 ± 0.01^Da^	1.27 ± 0.01^Ca^	1.20 ± 0.01^Ca^	1.05 ± 0.01^Ca^	1.10 ± 0.01^Bb^	1.02 ± 0.01^Ba^	0.88 ± 0.01^Bb^	0.94 ± 0.01^Ca^	0.89 ± 0.01^Ca^	0.74 ± 0.01^Da^	0.57 ± 0.01^Ba^	0.20 ± 0.01^Bab^

The means at the same frozen temperature with different uppercase letters (A–E) differ significantly (*p* < 0.05); the means at the same frozen time with different lowercase letters (a–d) differ significantly (*p* < 0.05).

### 3.3 TCA-soluble peptide

**Figure 2:**
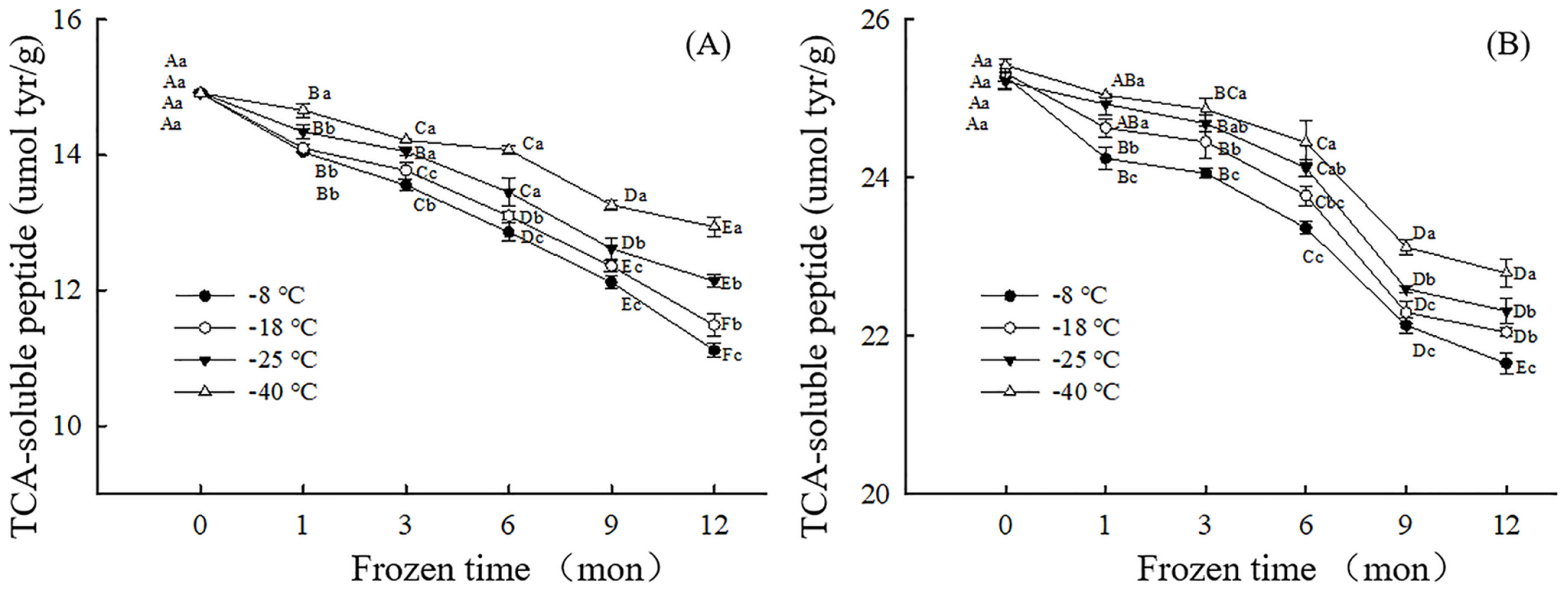
Influence of frozen storage temperature (°C) and time (mon) on the TCA-soluble peptide content of myofibrillar protein *in vitro* gastric **(A)** and small intestinal **(B)** digestion products.

The change in TCA-soluble peptide content can reflect the degree of protein digestion during frozen storage (25). The higher the TCA-soluble peptide content, the more digestible the protein. As shown in Figure 2, after 12 months of frozen storage, the TCA-soluble peptide content of the digested samples after simulated gastric and small intestinal digestion *in vitro* had decreased by 25.46% (−8°C), 22.99% (−18°C), 18.63% (−25°C), 13.23% (−40°C) and 14.37%(−8°C), 12.94% (−18°C),11.52% (−25°C), 10.35% (−40°C) respectively, compared to their corresponding initial values. The result showed that the lower the freezing temperature, the higher the content of TCA-soluble peptides and the easier the protein digestion. During simulated digestion *in vitro*, pepsin mainly recognizes amino or carboxyl groups at both ends of aromatic amino acids such as phenylalanine, tryptophan and tyrosine. At higher freezing temperature, free radical release rate is accelerated, tryptophan is easily oxidized by peroxy free radicals to produce stable kynuridine, and tyrosine free radical crosslinks to produce dityrosine. Therefore, the natural structure and content of amino acids at the recognition site of pepsin are changed, which reduces the sensitivity of pepsin hydrolysis (37, 38). This results in a decrease in TCA-soluble peptide content. However, under the condition of low temperature freezing storage, the protein structure and conformation are moderately developed, and the tryptophan and tyrosine buried in the protein are exposed, which can be well combined with pepsin, improve the digestion degree of the sample, and thus increase the TCA-soluble peptide content of the sample. Similarly, higher freezing temperatures accelerate the formation of oxidized aggregates and crosslinked products, which can alter the physical recognition site of trypsin, thereby reducing proteolytic sensitivity. In addition, at higher freezing temperature, lysine and arginine undergo a large degree of oxidative modification, resulting in changes in protease recognition sites, thus reducing the sensitivity of proteolysis and significantly reducing the content of TCA-soluble peptides after digestion. Fang et al. (39) noticed fewer cleavage sites for proteases and decreased TCA-soluble peptide content after *in vitro* digestion of surimi gels when the proteins were cross-linked by microbial transglutaminase.

### 3.4 Microscopic morphology

**Figure 3:**
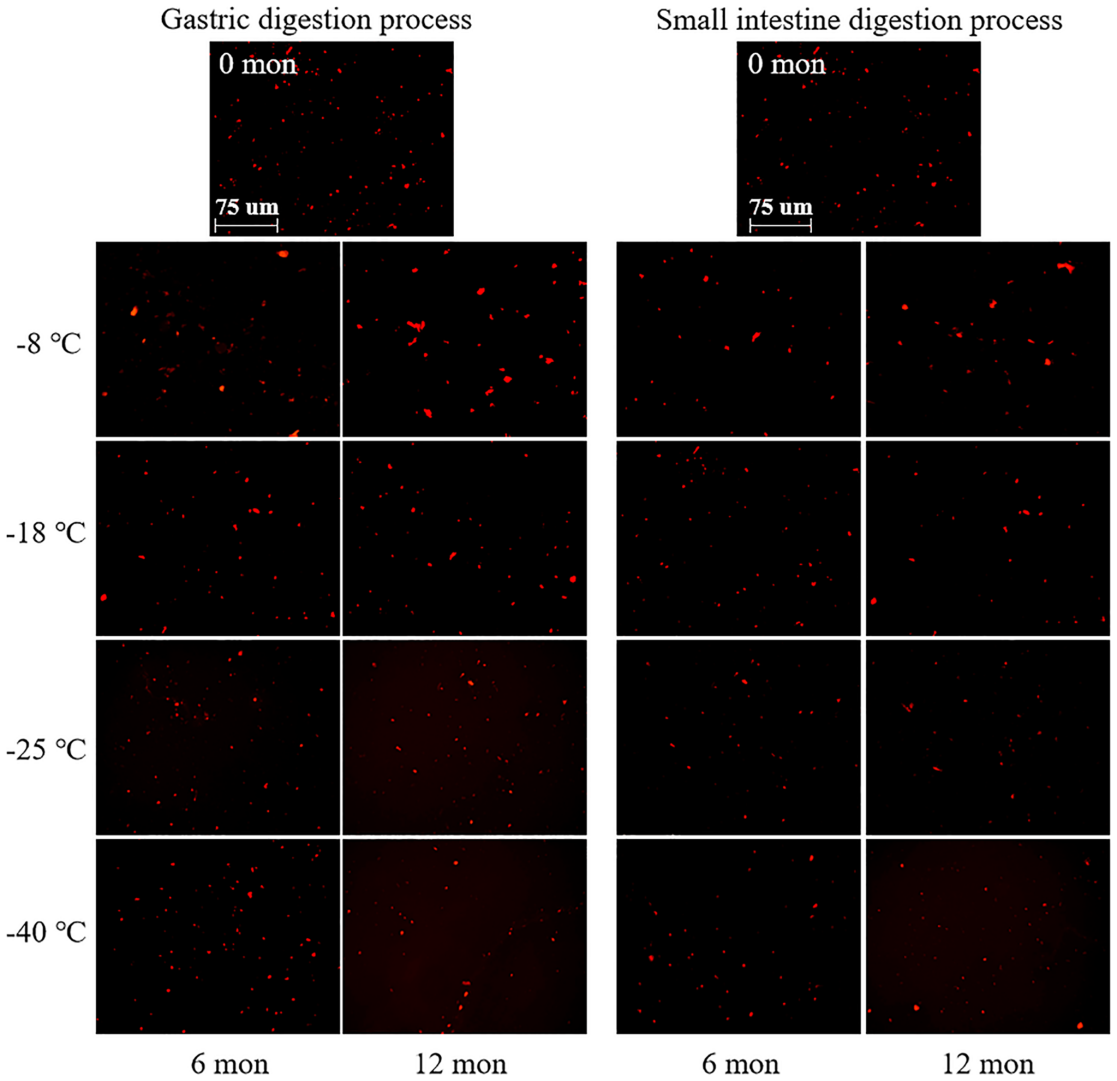
Influence of frozen storage temperature (°C) and time (mon) on the microstructure of myofibrillar protein *in vitro* digestion products.

CLSM was used to observe the microstructure of digested products after freezing for 6 and 12 months. As shown in Figure 3, the digested protein was stained with a bright red fluorescence by Nile blue. After digestion in the stomach and small intestine, the red droplets of fresh samples were dense and uniform distribution, and relatively small. This phenomenon indicated that fresh meat protein was easy to digest and absorb. And the TCA-soluble peptide content was the highest, which manifested a high digestibility. The microstructures exhibited different tendencies when the meat proteins were subjected to different frozen temperatures and storage durations. When the samples were stored at-8°C, the size and distribution of red droplets were nonhomogeneous, and the large droplets increased gradually with prolonged storage time. By contrast, the samples stored at-40°C showed well-distributed and relatively small red droplets. This result revealed that the pork muscle meat that had been subjected to long term high temperature frozen was difficultly to digest, which corresponded to low digestibility. The explanation for this phenomenon could be that proteins were less likely to denature and aggregate in samples stored at ultra-low temperature freezing (−40°C) compared to-8°C (40). At-40°C, the low extent of protein aggregation and denaturation meant that digestive enzymes could efficiently hydrolyze the protein into amino acids. Thus, the red fluorescent droplets were relatively uniformly distributed. At-8°C, however, protein aggregates were formed that were difficult to hydrolyze by digestive enzymes (pepsin and trypsin). Thus, large red droplets were observed by CLSM and a lower TCA-soluble peptide content was monitored. In addition, the interaction between disulfide bonds, dityrosine, and active carbonyl groups generated by protein oxidation promotes protein aggregation and binding, thus negatively affecting the rate of protein hydrolysis, resulting in relatively large red droplets after Nile blue staining (41). In related work, Bai et al. (14) found that repeated freezing and thawing of chicken meat was difficult to digest, and the red fluorescent droplets observed by CLSM became large.

### 3.5 Secondary structure

**Figure 4:**
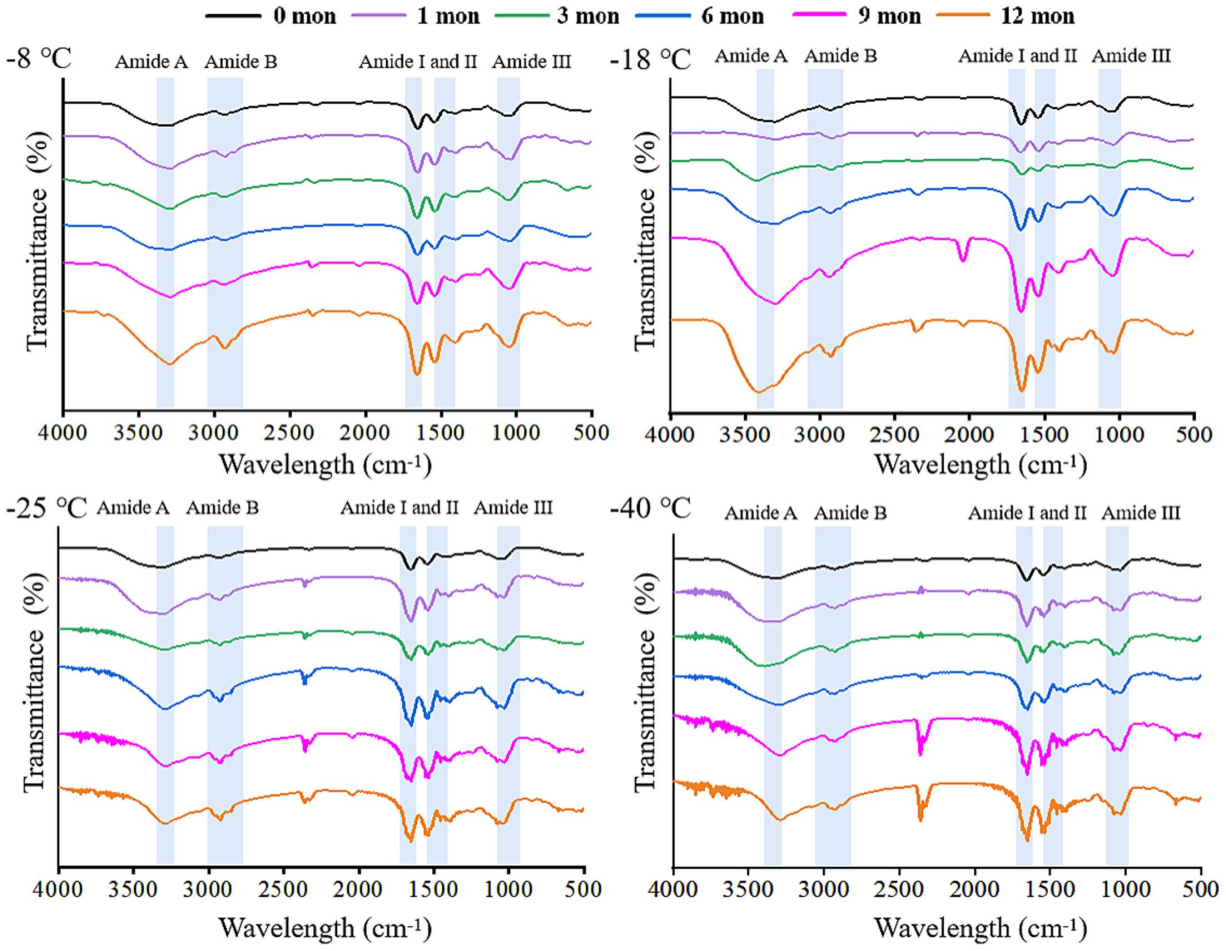
Influence of frozen storage temperature (°C) and time (mon) on the spectral of myofibrillar protein *in vitro* gastric digestion products.

**Figure 5:**
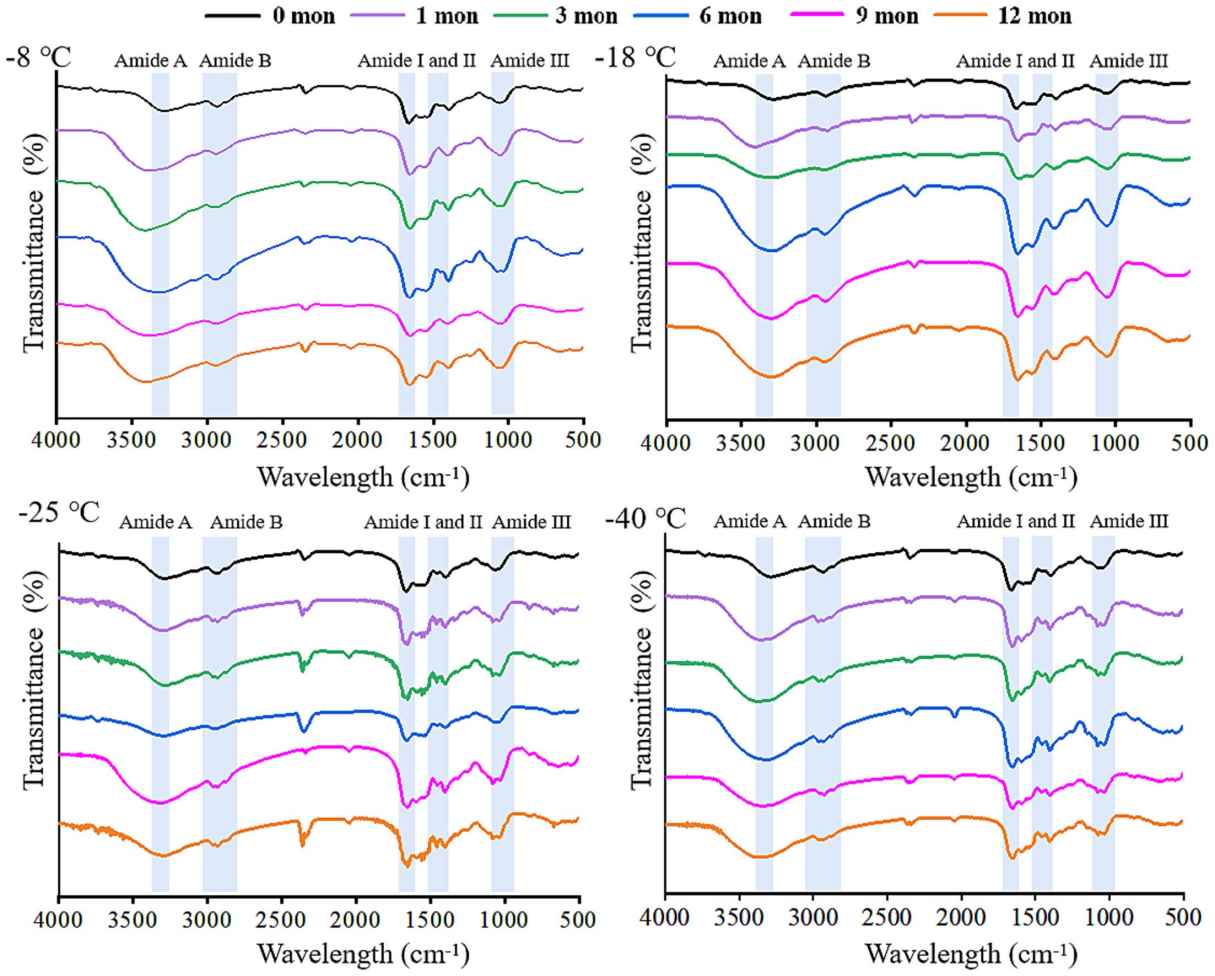
Influence of frozen storage temperature (°C) and time (mon) on the the spectral of myofibrillar protein *in vitro* small intestinal digestion product.

FTIR is a useful tool to monitor the secondary structure of digested products (42). The spectral changes of the products after digestion *in vitro* are shown in Figures 4, 5. There were five spectral regions in the FTIR of all samples: N-H stretching vibration of intermolecular hydrogen bonds (3200–3,600 cm^−1^, amide A), stretching of methylene asymmetric ester C-H in the side chain of proteins (2800–3,100 cm^−1^, amide B), C=O and C-N stretching vibration (1600–1700 cm^−1^, amide I), N-H bending and C-N and C-C stretching (1500–1,600 cm^−1^, amide II), and C-N and N-H stretching (1100–1,300 cm^−1^, amide III) (32, 43). With increased storage time, the shape of the peak gradually became narrower and sharper. The higher the frozen temperature, the more obvious the change in the shape of the peak. There was a red shift in the amide A and amide B bands, while the amide I, amide II, and amide III bands showed a blue shift. It could be that pepsin and trypsin mainly hydrolyzed the peptide bonds of Phe, Tyr, and Trp and the C-terminal bonds of Arg and Lys during digestion, which resulted in the exposure of C=O and N-H; hence, the observed shifts in the amide A, amide B, amide I, amide II, and amide III bands (44, 45).

The secondary structure of samples after digestion were further analyzed using the band of amide I and the amide I band in the FTIR included α-helix, β-sheet, β-turn, and random coil. Tables 3, 4 shows the change in the secondary structure content of the digestive products after stomach and small intestine digestion, respectively. At the same freezing temperature, the contents of the α-helix and β-turn decreased and the contents of B-fold and random curling increased after pepsin digestion with the extension of freezing time. Then, after trypsin digestion, with the extension of frozen storage time, the α-helix and β-turn contents of samples were futher decreased, while the β-sheet and random coil contents were futher increased. At the end of freezing storage at-8°C, the contents of α-helix, β-sheet, β-turn and rrandom coil reached 34.19, 25.38, 5.02 and 35.13%, respectively. This is because after the enzymatic hydrolysis of protease, the spatial structure and conformation of protein molecules are unfolded, resulting in the destruction of the ordered structure of α-helix and β-turn, and the gradual transformation into β-sheet and random coils (25, 46). In addition, The decrease in the α-helix content and an increase in the random coil content was accompanied by the decrease in the TCA-soluble peptide and protein digestibility. Furthermore, the β-sheet structure was negative correlation with protein digestibility. The higher the percentage of the β-sheet structures, the lower TCA-soluble peptide. Bai et al. (14) showed that protein cross-linking and aggregation in the frozen storage process reduced the cleavage site and chemical reaction site of pepsin, ultimately leading to changes in the secondary structure of protein digestion products. Zhou et al. (47) found that the alpha-helix content was reduced, indicating that the secondary structure of the protein was further developed after trypsin digestion. Zhang et al. (48) believed that the change of protein helical structure was accompanied by protein aggregation, which masked the recognition sites and thus affected the digestion process of protease. Du et al. (49) reported that the decrease in α-helix content was related to the reduction in TCA-soluble peptide content. Furthermore, the β-folded structure contains many hydrogen bonds, which hinder the digestion reaction and therefore was negatively correlated with the degree of protein digestion.

**Table 3:** Influence of frozen storage temperature (°C) and time (mon) on the secondary structure contents of myofibrillar protein *in vitro* gastric digestion product.

Temperature (°C)	Time (mon)	α-helix (%)	β-sheet (%)	β-turn (%)	Random coil (%)
−8	0	51.77 ± 1.11^Aa^	17.71 ± 0.02^Ca^	11.88 ± 0.36^Aa^	18.78 ± 0.57^Da^
	1	50.36 ± 0.10^Aa^	18.06 ± 1.23B^Ca^	11.63 ± 0.30^Aa^	20.47 ± 1.50^Da^
	3	47.14 ± 0.45^Bb^	18.45 ± 1.44^BCa^	9.92 ± 0.31^Bb^	24.03 ± 1.19^Ca^
	6	45.36 ± 1.32^Ba^	20.18 ± 0.22^BCa^	8.53 ± 0.18^Cc^	26.14 ± 0.37^Ca^
	9	40.26 ± 1.12^Cb^	20.95 ± 0.79^ABa^	7.28 ± 0.16^Dd^	30.57 ± 0.76^Ba^
	12	38.26 ± 1.46^Cb^	22.31 ± 1.45^Aa^	7.09 ± 0.196^Dc^	32.85 ± 0.49^Aa^
−18	0	51.77 ± 1.11^Aa^	17.71 ± 0.02^Ea^	11.88 ± 0.36^Aa^	18.78 ± 0.57^ABa^
	1	50.66 ± 0.68^ABa^	17.94 ± 0.05^Da^	11.44 ± 0.42^Aa^	20.19 ± 1.41^Ba^
	3	48.14 ± 1.23^BCab^	18.12 ± 0.11^Ca^	10.28 ± 0.06^Ba^	23.42 ± 0.57^Ca^
	6	45.58 ± 1.34^CDa^	19.18 ± 0.05^Bb^	9.16 ± 0.19^Cb^	26.35 ± 0.20^Da^
	9	44.26 ± 0.28^DEa^	20.15 ± 0.14^Aab^	7.93 ± 0.34^Dc^	27.84 ± 0.42^Eb^
	12	41.26 ± 1.34^Eab^	20.32 ± 0.02^Ab^	7.28 ± 0.11^Ec^	31.21 ± 0.67^Fb^
−25	0	51.77 ± 1.11^Aa^	17.71 ± 0.02^Fa^	11.88 ± 0.36^Aa^	18.78 ± 0.57^Ea^
	1	50.76 ± 1.23^Aa^	17.86 ± 0.05^Ea^	11.51 ± 0.24^Aa^	20.00 ± 1.41^Ea^
	3	49.52 ± 0.89^ABa^	18.08 ± 0.04^Da^	10.41 ± 0.08^Ba^	22.01 ± 1.22^Dab^
	6	46.58 ± 2.01^BCa^	18.43 ± 0.07^Cc^	9.23 ± 0.27^Cab^	25.49 ± 0.54^Cab^
	9	45.56 ± 0.07^CDa^	18.85 ± 0.11^Bbc^	8.46 ± 0.18^Db^	27.20 ± 0.35^Bb^
	12	42.71 ± 0.09^Da^	19.78 ± 0.05^Ab^	7.78 ± 0.09^Eb^	29.63 ± 0.52^Ac^
−40	0	51.77 ± 1.11^Aa^	17.71 ± 0.02^Ea^	11.88 ± 0.36^Aa^	18.78 ± 0.57^Ea^
	1	51.36 ± 0.78^Aa^	17.96 ± 0.02^Da^	11.18 ± 0.79^ABa^	19.23 ± 1.29^Ea^
	3	50.14 ± 0.44^ABa^	18.02 ± 0.05^Da^	10.55 ± 0.59^BCa^	21.15 ± 0.23^Db^
	6	48.36 ± 1.11^BCa^	18.36 ± 0.08^Cc^	9.58 ± 0.21^Da^	23.25 ± 0.61^Cb^
	9	6.720 ± 1.33^CDa^	18.75 ± 0.05^Bc^	9.01 ± 0.10^DEa^	25.36 ± 0.80^Bc^
	12	44.28 ± 1.01^Da^	19.41 ± 0.08^Ab^	8.28 ± 0.40^Fa^	28.33 ± 0.36^Ad^

The means at the same frozen temperature with different uppercase letters (A–E) differ significantly (*p* < 0.05); the means at the same frozen time with different lowercase letters (a-d) differ significantly (*p* < 0.05).

**Table 4:** Influence of frozen storage temperature (°C) and time (mon) on the secondary structure contents of myofibrillar protein *in vitro* small intestinal digestion product.

Temperature (°C)	Time (mon)	α-helix (%)	β-sheet (%)	β-turn (%)	Random coil (%)
−8	0	48.31 ± 1.22^Aa^	21.11 ± 1.01^Ca^	10.23 ± 0.74^Aa^	21.61 ± 0.77^Ca^
	1	47.21 ± 1.79^Aa^	21.46 ± 1.20^Ca^	10.17 ± 0.83^Aa^	22.57 ± 2.03^Ca^
	3	43.07 ± 1.20^Bb^	21.85 ± 1.15^BCa^	8.05 ± 0.20^Bb^	26.86 ± 0.59^Ba^
	6	41.29 ± 1.31^Bb^	23.58 ± 1.02^ABa^	6.22 ± 0.49^Cb^	28.82 ± 1.21^Ba^
	9	36.19 ± 1.51^Cc^	24.35 ± 1.18^Aa^	5.21 ± 0.34^CDc^	34.06 ± 1.12^Aa^
	12	34.19 ± 1.39^Cc^	25.38 ± 0.82^Aa^	5.02 ± 0.17^Db^	35.13 ± 0.65^Aa^
−18	0	47.70 ± 1.23^Aa^	21.11 ± 1.01^Ba^	10.23 ± 0.74^Aa^	21.61 ± 0.77^Da^
	1	46.59 ± 1.27^Aa^	20.97 ± 0.85^Ba^	9.97 ± 1.13^Aa^	22.61 ± 1.48^Da^
	3	44.28 ± 1.01^ABab^	21.93 ± 1.13^ABa^	8.17 ± 0.11^Bb^	25.95 ± 1.02^Cab^
	6	41.48 ± 1.45^BCb^	22.48 ± 0.26^ABab^	6.81 ± 0.99^BCb^	28.63 ± 1.58^Bab^
	9	40.19 ± 1.36^CDb^	23.88 ± 1.63^Aa^	5.89 ± 0.46^Cbc^	30.32 ± 0.61^Bb^
	12	37.16 ± 1.25^Db^	23.95 ± 1.17^Aab^	5.58 ± 0.61^Cab^	33.61 ± 2.05^Aab^
−25	0	47.70 ± 1.52^Aa^	21.11 ± 1.01^Aa^	10.23 ± 0.74^Aab^	21.61 ± 0.77^Da^
	1	46.69 ± 1.21^Aa^	21.24 ± 1.44^Aa^	9.71 ± 0.32^Aa^	22.60 ± 0.36^Da^
	3	45.45 ± 1.22^ABa^	21.48 ± 0.29^Aab^	8.46 ± 0.21^Bab^	24.56 ± 1.11^Cbc^
	6	42.51 ± 1.32^Bab^	21.83 ± 0.42^Ab^	7.39 ± 0.43^BCa^	28.41 ± 0.50^Bab^
	9	42.36 ± 0.75^Bab^	22.25 ± 1.33^Ab^	6.48 ± 0.79^CDab^	29.44 ± 1.31^Bbc^
	12	38.63 ± 1.11^Cab^	23.18 ± 0.51^Ab^	5.63 ± 0.78^Dab^	32.12 ± 1.33^Abc^
−40	0	47.63 ± 0.31^Aa^	21.11 ± 1.11^Ba^	10.23 ± 0.74^Aa^	21.61 ± 0.77^Ea^
	1	47.29 ± 1.51^Aa^	21.36 ± 0.53^Ba^	9.71 ± 0.55^ABa^	22.04 ± 0.45^Ea^
	3	46.07 ± 1.22^ABa^	21.42 ± 0.42^Bb^	8.72 ± 0.13^Ba^	23.86 ± 0.86^Dc^
	6	44.29 ± 1.53^ABa^	21.77 ± 0.72^ABb^	7.39 ± 0.79^Ca^	26.52 ± 1.22^Cc^
	9	42.62 ± 1.25^BCa^	22.15 ± 0.32^ABb^	7.02 ± 0.47^CDa^	28.26 ± 0.98^Bc^
	12	40.18 ± 1.55^Ca^	22.81 ± 0.53^Ab^	6.17 ± 0.55^Da^	30.51 ± 0.48^Ac^

The means at the same frozen temperature with different uppercase letters (A–E) differ significantly (*p* < 0.05); the means at the same frozen time with different lowercase letters (a–d) differ significantly (*p* < 0.05).

### 3.6 Tertiary structure

**Figure 6:**
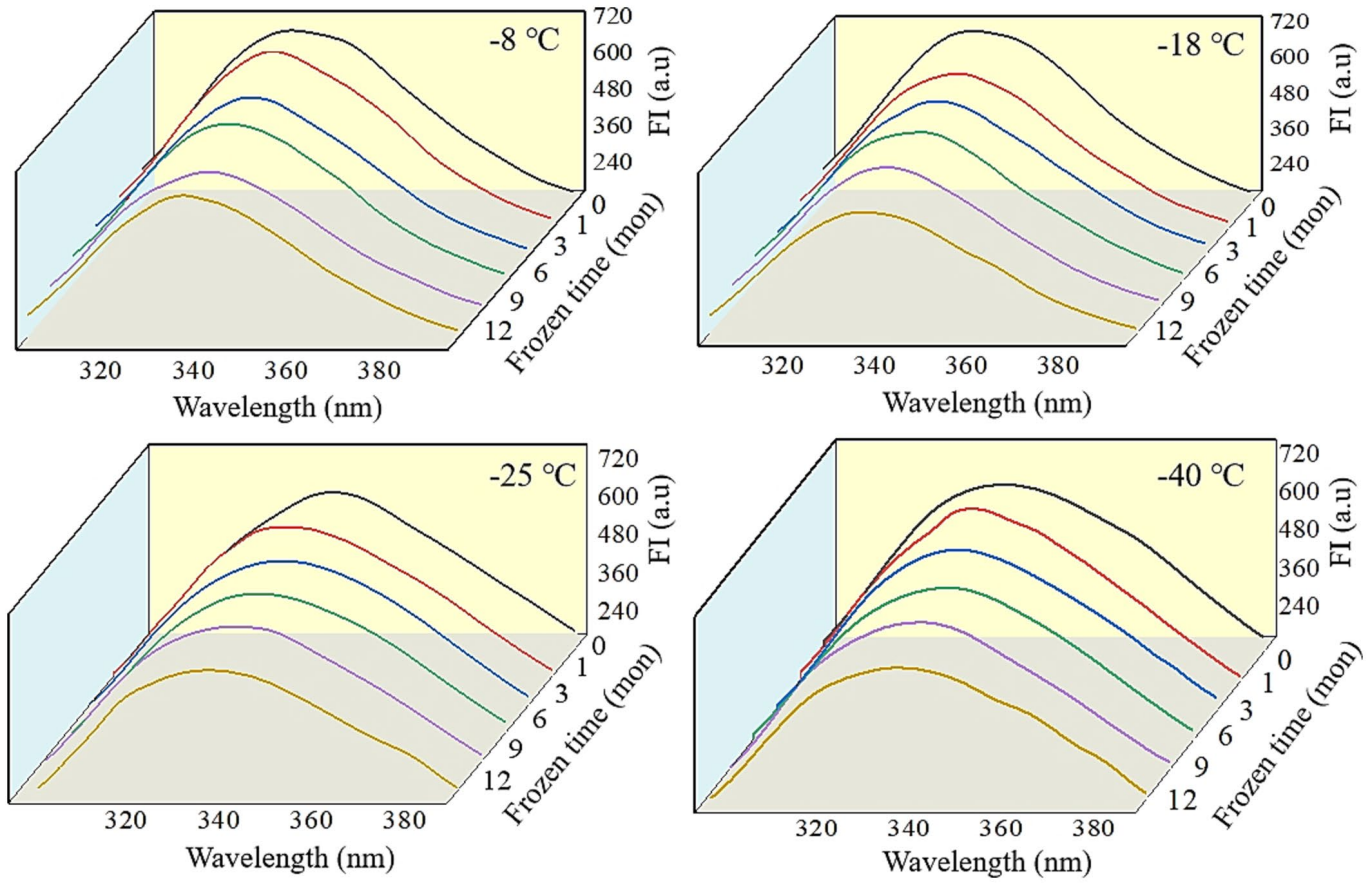
Influence of frozen storage temperature (°C) and time (mon) on the tertiary structure of myofibrillar protein *in vitro* gastric digestion products.

**Figure 7:**
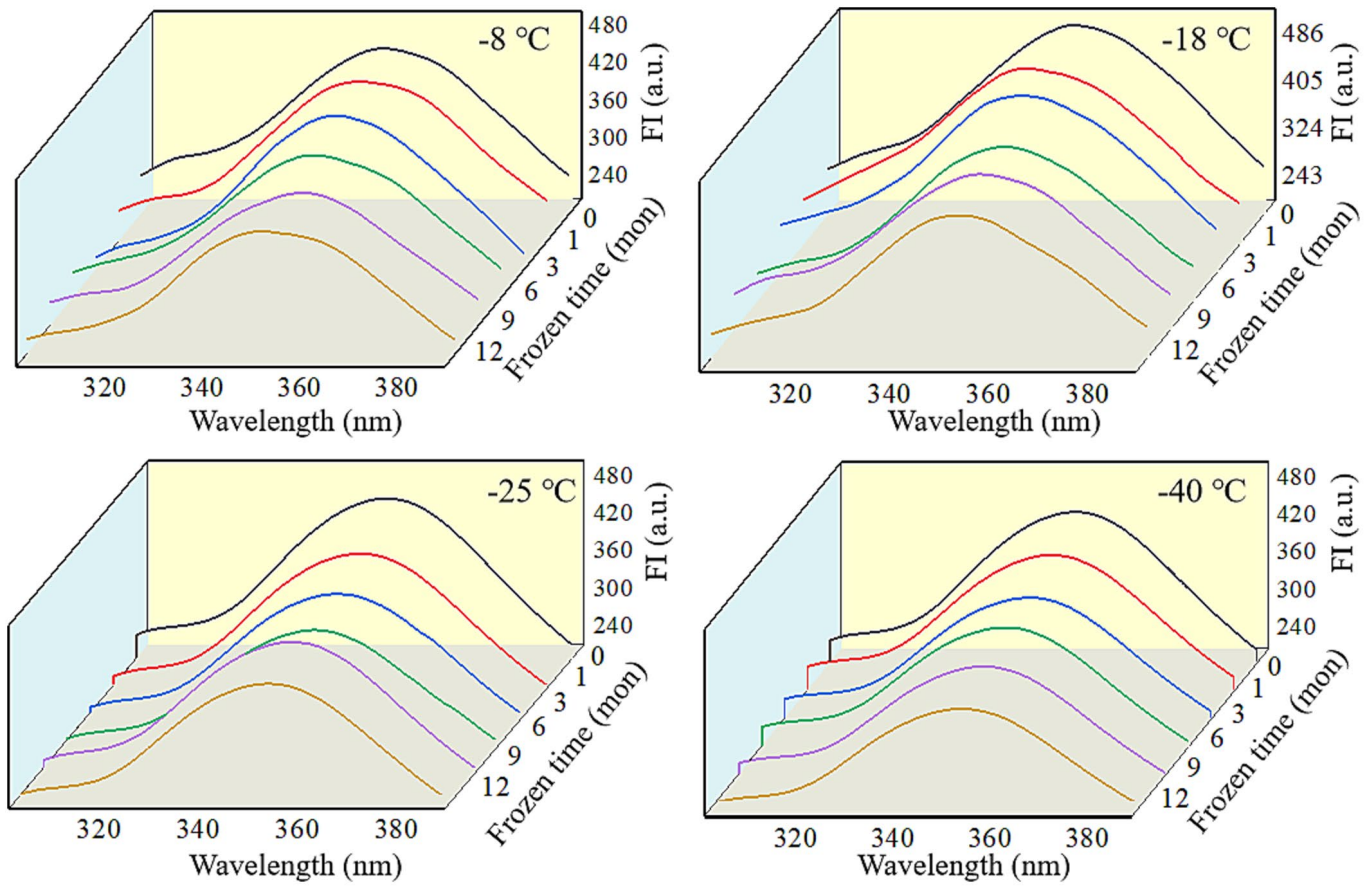
Influence of frozen storage temperature (°C) and time (mon) on the tertiary structure of myofibrillar protein *in vitro* small intestinal digestion products.

Fluorescence spectra can reflect the changes in the tertiary conformation of MP after *in vitro* digestion (50). As shown in Figures 6, 7, after simulated gastrointestinal digestion, the maximum fluorescence wavelength (λ_max_) had red-shifted. For samples subjected to 12 months of frozen storage, λ_max_ shifted to 349 nm (−8°C), 348 nm (−18°C), 347 nm (−25°C), and 346 nm (−40°C) after simulated gastric digestion *in vitro*, and to 361 nm (−8°C), 359 nm (−18°C), 357 nm (−25°C), and 356 nm (−40°C) after simulated small intestinal digestion *in vitro*. In addition, the FI of digested products was significantly decreased (*p* < 0.05). During the *in vitro* simulated digestion, pepsin mainly hydrolyzes the peptide bonds of aromatic amino acids (51). During frozen storage, protein unfolding causes the exposure and oxidation of tryptophan to produce stable kynurenine, resulting in a decrease in the amount of amino acids hydrolyzed by pepsin and a red shift in the FTIR spectrum (52). Moreover, protein cross-linking and aggregation could result in fluorescence quenching (53). Liu et al. (54) found that the FI was usually related to the energy transfer from Tyr to Trp and adjacent fluorescence quenching groups. When MP was hydrolyzed by pepsin and trypsin, the energy transfer of Tyr to Trp was increased, and the amount of fluorescence quenching groups was decreased. Thus, a decline in FI was observed.

## 4 Conclusion

This study assessed the influence of frozen storage time and temperature on the basic components and *in vitro* digestive characteristics of pork. The moisture, crude protein, and fat content decreased significantly with prolonged storage and increased frozen storage temperature. Meanwhile, the amino acids (such as Glu, Asp., and Lys) in muscle significantly increased in the first 6 months and decreased afterward. The results of *in vitro* digestion showed that the TCA-soluble peptide, α-helix, and β-turn contents and the FI of samples significantly decreased, while the β-sheet and random coil contents and λ_max_ significantly increased. Therefore, longer frozen storage and higher temperature storage accelerated the decline in the basic components of pork, reduced the degree of protein digestion, and destroyed the secondary and tertiary structure of digested products.

## Data Availability

The raw data supporting the conclusions of this article will be made available by the authors, without undue reservation.
